# Ultrastructure of three species of *Tryblionella* (Bacillariophyceae) found in freshwater habitats in Hunan Province, China, including descriptions of two new species

**DOI:** 10.3897/phytokeys.278.204336

**Published:** 2026-08-25

**Authors:** Ya-Lun Ma, Can-Ping Yu, Bing Liu, Patrick Rioual, Yi-Dan Ma, Xiao-Yu Jiang

**Affiliations:** 1 School of Life Sciences, Jishou University, Jishou 416000, China State Key Laboratory of Lithospheric and Environmental Coevolution, Institute of Geology and Geophysics, Chinese Academy of Sciences Beijing China; 2 State Key Laboratory of Lithospheric and Environmental Coevolution, Institute of Geology and Geophysics, Chinese Academy of Sciences, P.O. box 9825, Beijing 100029, China School of Life Sciences, Jishou University Jishou China

**Keywords:** Freshwater diatom, marginal ridge, new species, *
Tryblionella
*, ultrastructure

## Abstract

Two diatom samples collected from two freshwater habitats in Hunan Province, China, were studied using light microscopy and scanning electron microscopy. Three *Tryblionella* species were found; one was identified as *T. calida* (Grunow) Stroganov et al., and the other two species are established here as new species: *T. dongtingensis***sp. nov**. and *T. hunanensis***sp. nov**. These two new species are morphologically allied to *T. calida* because they possess the following unique combination of characters: 1) a more or less undulate valve face from side to side; 2) transapical ribs; 3) uniseriate striae with rounded, rimmed inner openings of areolae; 4) a longitudinal row of bar-like ribs connecting the raphe canal wall on both the valve side and the distal mantle side; 5) a marginal ridge; and 6) squat, sometimes apically elongated fibulae. This group around *T. calida* is different from the group around *T. apiculata* W. Gregory, which possesses an axial sternum and different stria and areola structures. Additional details of the complex marginal ridges in *Tryblionella* are revealed and discussed in this paper. Whether the group around *T. calida* is a monophyletic group and possesses a synapomorphy cannot currently be resolved, and further investigations are needed.

## Introduction

Smith (1853) established the diatom genus *Tryblionella* based on six new species within this new genus. Round et al. (1990) listed the shapes and structures of both the fibulae and the marginal ridge, stria types, the presence or absence of a sternum, and the undulation of the valve face from side to side as a diagnostic combination of characters for the genus *Tryblionella*. Recently, Mann et al. (2021) reassessed the diatom family Bacillariaceae and concluded that *Tryblionella* is non-monophyletic. For the time being, because the circumscription of a monophyletic *Tryblionella* has been expanded, the genus is referred to as *Tryblionellasensu lato* (e.g., Guiry in Guiry and Guiry 2026). Since 1990, only four species of *Tryblionella* have been described as new species (Witkowski et al. 2016; Bertolli et al. 2019; Ma et al. 2025), and none of them have been found in freshwater habitats. The frustule ultrastructure of most known species of *Tryblionella* has not been adequately documented, and fine details of the fibulae and marginal ridge are very often lacking (Ma et al. 2025).

In China, 10 species of *Tryblionellasensu lato* have been listed for the freshwater diatom flora (Wang 2018). In the marine realm, *T. gaoana* Witkowski & Li was described in the littoral zone of the Bohai Sea (Witkowski et al. 2016). Ma et al. (2025) documented in detail the ultrastructure of three species of *Tryblionella* found in the brackish water of Lake Qinghai, China. In particular, they clearly illustrated and explained the ultrastructure of the marginal ridge and distal mantle for *T. apiculata* W. Gregory and *T. levidensis* W. Smith and provided valuable new insights into *Tryblionella*. In the same year that the work on Lake Qinghai was published, the marginal ridge was also reviewed and discussed by Olszyński et al. (2025). Thus, the marginal ridge, which was included as one of the features of *Tryblionella* by Round et al. (1990) but neglected by later researchers, has recently attracted increasing attention from researchers.

In Hunan Province, China, freshwater habitats are abundant and include many rivers as well as Dongting Lake, the second-largest freshwater lake in China. The diatom flora of these freshwater habitats has been continuously studied by Dr. Bing Liu and collaborators, and their studies have revealed that there is a relatively high diversity of diatoms in Dongting Lake and the Xiang River (e.g., Liu et al. 2021; Long et al. 2021; Xiang et al. 2024; Yi et al. 2024; Zheng et al. 2024). The present paper describes three species of *Tryblionellasensu lato* collected from Dongting Lake and the Xiang River. The aim is to demonstrate clearly the ultrastructure of *T. calida* and to establish two species as new to science.

## Materials and methods

A diatom sample was collected from the surface sediments in the littoral zone of East Dongting Lake (29°19'7"N, 112°47'32"E, 39 m a.s.l.), Hunan Province, China, on 5 March 2023. Specimens of three species [*Tryblionella calida* (Grunow) Stroganov et al., *T. dongtingensis* sp. nov., and *T. hunanensis* sp. nov.] were found in this diatom sample. The field sampling method followed that described by Yi et al. (2024).

Another diatom sample was collected from the Yongle River (26°55'08"N, 113°35'85"E, 119 m a.s.l.), a tributary of the Xiang River, the largest river in Hunan Province, China, on 24 March 2024. Specimens of *Tryblionella calida* and *T. hunanensis* sp. nov. were also found in this diatom sample. The field sampling method followed that described by Zheng et al. (2024).

The methods of processing the two diatom samples above for microscopic examination, preparing permanent slides for light microscopy (LM) observations, and preparing specimens for scanning electron microscopy (SEM) observations followed those described by Liu (2023). The slides are deposited in the Herbarium of Jishou University, Hunan, People’s Republic of China (JIU) (herbarium acronyms follow Index Herbariorum: http://sweetgum.nybg.org/science/ih/).

Diatom terminology largely follows Round et al. (1990) and Ma et al. (2025). In external valvar view, transversal ribs are defined here as the ribs parallel to the transverse cross-section, and bar-like ribs are defined here as the short ribs connecting the raphe canal wall on both the valve side (e.g., Fig. 2) and proximal mantle side (e.g., Fig. 3B–D) in this paper.

**Figure 1.**
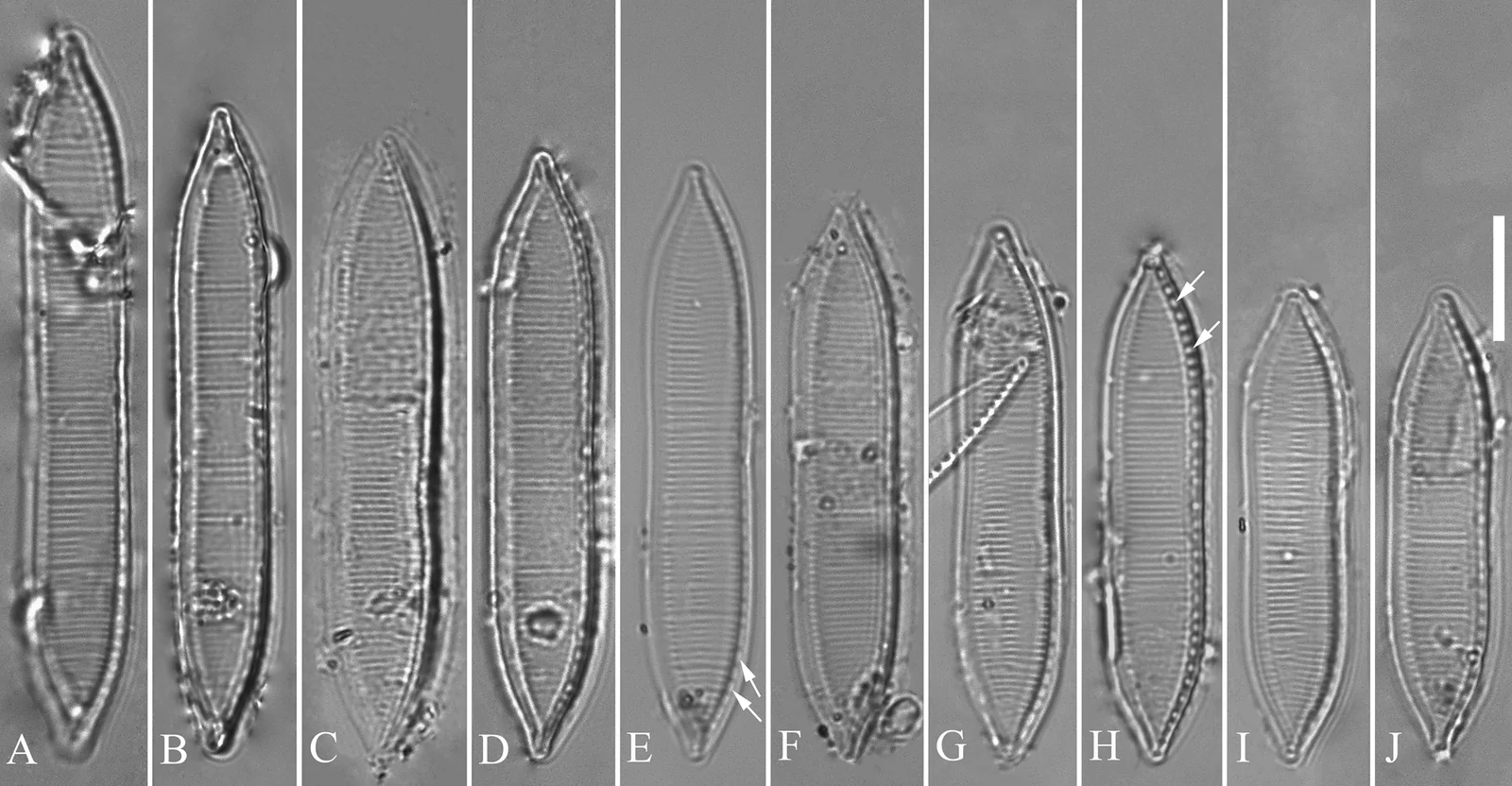
*Tryblionella calida*, LM. (**A–J**). Ten valves showing a size-diminution series; note the transapical ribs extending across the valve surface, absence of an axial sternum, and rectangular to square fibulae (e.g., **E**, **H**, arrows). Scale bar: 10 μm (**A–J**).

**Figure 2.**
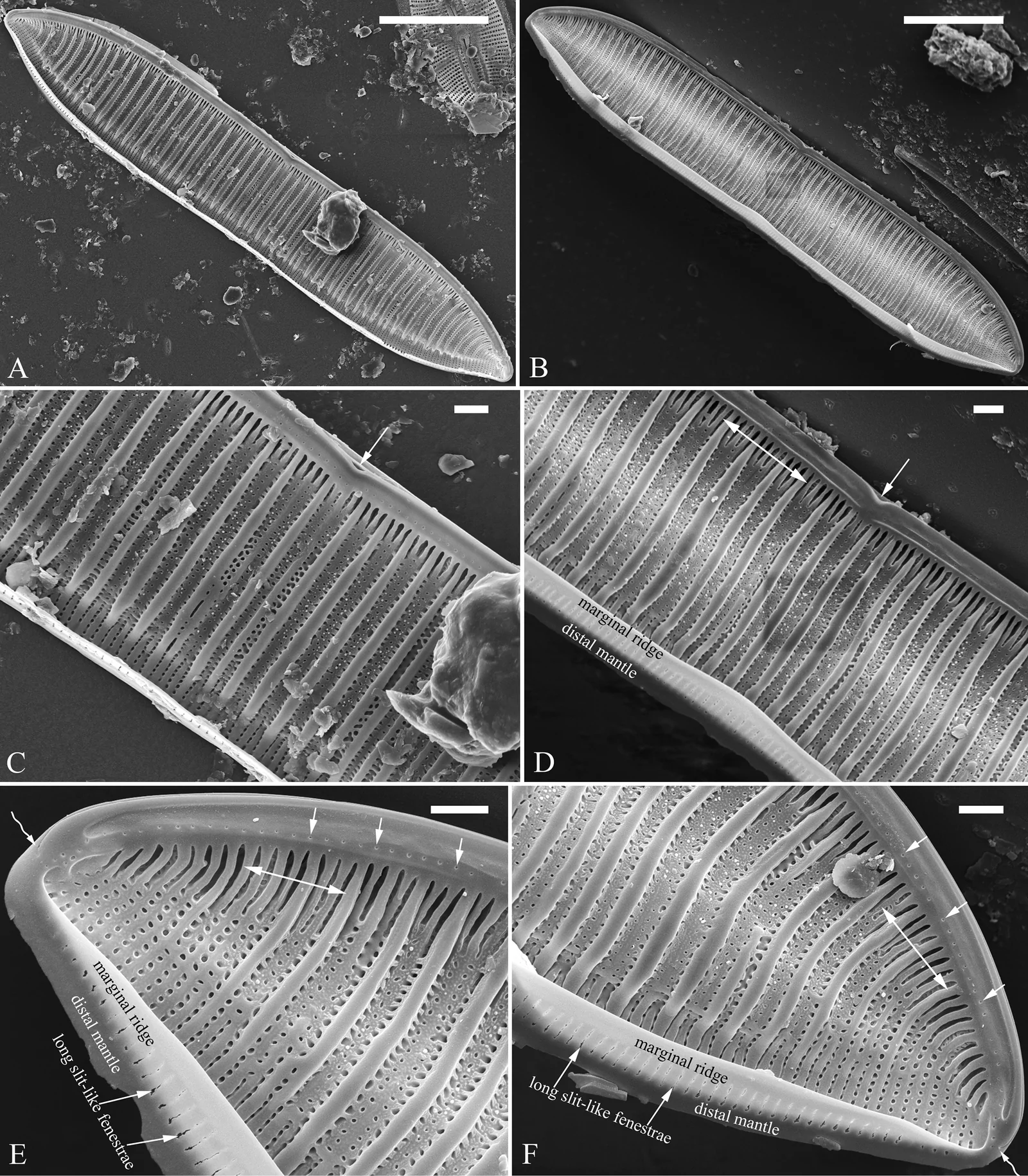
*Tryblionella calida*, external view, SEM. **A**, **B**. Two complete valves; note the high marginal ridge, transapical ribs extending across the valve surface, and absence of an axial sternum. **C**. Detail of the middle part from **A**; note the depressed central nodule (arrow). **D–F**. Details from **B**; note the shallow distal mantle, the long slit-like fenestrae (labeled in **E**, **F**) at the base of the marginal ridge, the smooth and straight upper margin of the marginal ridge, a longitudinal row of short bar-like ribs connecting the raphe canal wall (double-headed arrows), and a row of poroids produced in the raphe canal wall on the side of the valve surface (arrows). Scale bars: 10 μm (**A**, **B**); 1 μm (**C–F**).

**Figure 3.**
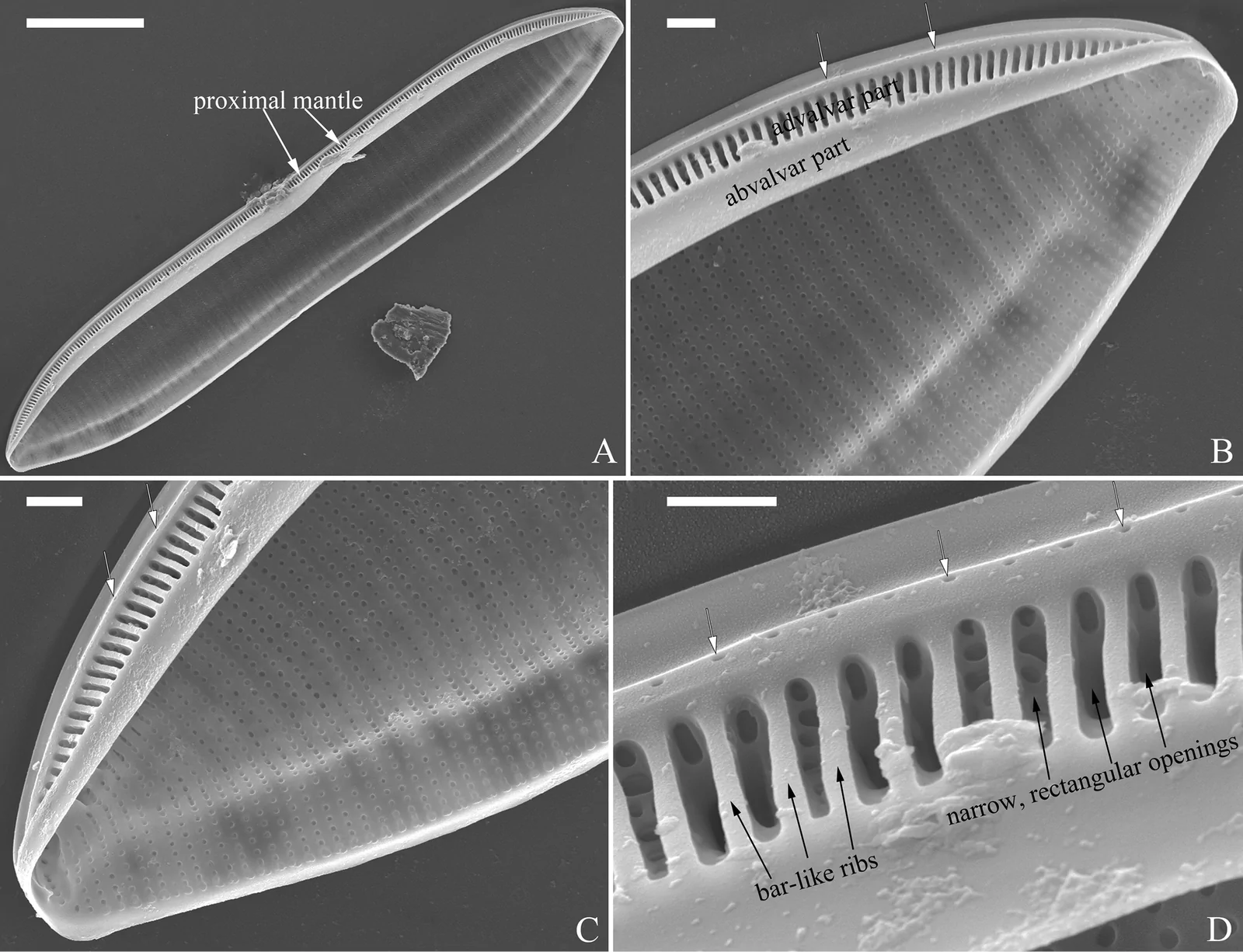
*Tryblionella calida*, internal view, proximal mantle aspect, SEM. **A**. A complete valve; note that the markedly deep proximal mantle hides the fibulae. **B–D**. Details from **A**; note the rimmed, rounded inner openings of areolae; the proximal mantle is composed of two different parts: an advalvar part (composed of bar-like ribs and narrow, rectangular openings, labeled in **D**) and an abvalvar part (hyaline without ornamentation); and a row of poroids is produced in the raphe canal wall on the side of the proximal mantle (arrows). Scale bars: 10 μm (**A**); 1 μm (**B**, **C**); 0.5 μm (**D**).

## Results

### Tryblionella calida

Taxon classificationPlantaeBacillarialesBacillariaceae

(Grunow) Stroganov

AB026FBB-AFC4-5B74-B458-DB555D929ABB

Figs 1, 2, 3, 4

#### Description.

***LM*** (Fig. 1). Valves linear-lanceolate with wedge-shaped, slightly protruding apices. Valve dimensions (*n* = 17): length range 38–61 μm, width range 8.6–10.6 μm at the constricted middle part. Valve surfaces only slightly undulate from side to side, appearing almost flat. Axial sternum absent. Transapical ribs parallel, continuous across valve surface, 15–17 in 10 μm. Raphe system eccentric, located along one side of valve. Fibulae visible, short, rectangular to square (e.g., Fig. 1E, H, arrows), 9–10 in 10 μm. Ratio of transapical-ribs density to fibula density ca. 1.6.

**Figure 4.**
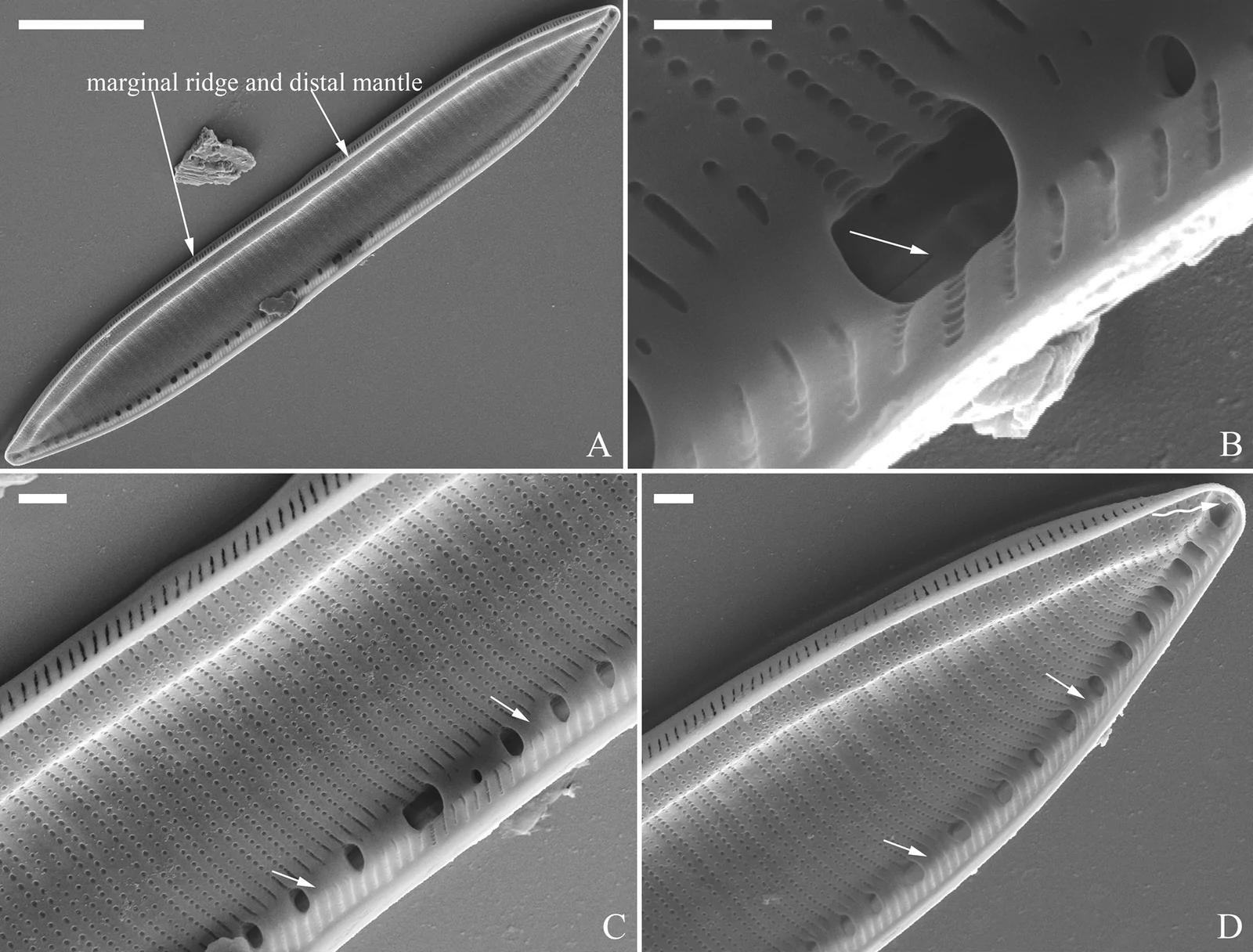
*Tryblionella calida*, internal view, SEM. **A**. A complete valve; note the marginal ridge and its long slit-like fenestrae and the shallow distal mantle. **B**, **C**. Details of the middle part from **A** showing the straight inner central raphe fissure (**B**, arrow), central nodule (helictoglossa), and non-striated fibulae, sometimes apically elongated (**C**, two arrows). **D**. Apical details from **B**; note the rounded inner openings of areolae, squat fibulae, sometimes apically elongated (two arrows), and a small helictoglossa (curved arrow). Scale bars: 10 μm (**A**); 0.5 μm (**B**); 1 μm (**C**, **D**).

***SEM*** (Figs 2, 3, 4). Transapical ribs elevated, continuously across valve surface (Fig. 2A, B). Striae uniseriate, 34–36 in 10 μm (measured on SEM images, *n* = 3). Marginal ridge is a high standing silica lamella, composed of a bottom part with long slit-like fenestrae (labelled in Fig. 2E, F) and a top part, smooth with a plain margin (Fig. 2). Distal mantle shallow, lacking ornamentations (Figs 2, 4A, C, D). Central nodule present (Fig. 2C, D, arrow; Fig. 4B, arrow), distal raphe fissures curved (Fig. 2E, F, curved arrows). A longitudinal row of short bar-like ribs is produced along the raphe canal wall from pole to pole (Fig. 2D–F, double-headed arrows). Row of poroids is produced in the raphe canal wall on the side of valve surface (Fig. 2E, F, arrows). Inner openings of areolae rounded, rimmed. Proximal mantle very deep so that it often hides the short fibulae (Fig. 3A). Proximal mantle composed of two parts: advalvar part (composed of bar-like ribs and narrow, rectangular openings, labelled in Fig. 3D), and abvalvar part (hyaline without ornamentations, labelled in Fig. 3B). A row of poroids produced in the raphe canal wall on the side of the proximal mantle (Fig. 3B–D, arrows). Inner central raphe fissure straight, central nodule helictoglossa-like (Fig. 4B, arrow). Fibulae squat, some apically elongated (Fig. 4C, D, arrows). Helictoglossae small (Fig. 4C, D, curved arrows).

#### Comments.

The basionym of *Tryblionella calida* is *Nitzschia calida* Grunow (in Cleve and Grunow 1880). Grunow’s protologue, written in German, reads: “*N. calida* Grunow. Klein, Schaalen linear länglich, meist in der Mitte schwach verengt, mit keilförmigen, schwach vorgezogenen Spitzen. Längsfalte breit, sehr flach, oft kaum sichtbar. Querstreifen 17–19 in 0.01 mm., Kielpunkte klein, rund, circa 10 in 0.01 mm. Länge 0.034–0.043 mm., Schaalenbreite 0.009–0.01 mm. In den Thermen von Ofen in Ungarn. […]” (Cleve and Grunow 1880, p. 75). The specimens examined fit Grunow’s protologue in valve morphology (e.g., especially the almost flat valve face), overlapping valve sizes (38–61 μm vs. 34–43 μm in length, 8.6–10.6 μm vs. 9–10 μm in width), overlapping density of transapical ribs (15–17 in 10 μm vs. 17–19 in 10 μm), overlapping fibula density (9–10 in 10 μm vs. 10 in 10 μm), and their occurrence in freshwater habitats.

Outside Europe, Kociolek (2011) provided observations of *T. calida* based on specimens collected from America and provided the following description: “[…] A narrow, indistinct fold runs along the longitudinal axis of the valve. The fold is barely undulated or may even appear not to rise or taper from the main valve surface. […].” Thus, Kociolek also noted the almost flat valve faces in *T. calida*.

SEM observations of *T. calida* are available in Metzeltin et al. (2005, plate 109) and Bertolli et al. (2020, Fig. 5D–G), but these studies provided only low-magnification images and did not show many details of the frustule ultrastructure. In this paper, the ultrastructure of its marginal ridge, proximal and distal mantles, and fibulae is described and illustrated in detail for the first time. Thus, based on the protologue and the observations presented here, the following diagnostic criteria are proposed for *T. calida*. These include a linear-lanceolate valve outline with wedge-shaped, slightly protruding apices, a very slightly undulate valve surface, elevated transapical ribs continuous across the valve face and short bar-like ribs connecting the raphe canal wall, uniseriate striae, squat and sometimes apically elongated fibulae, and a high marginal ridge composed of a bottom part with elongated slit-like fenestrae and a solid top part with a plain margin.

**Figure 5.**
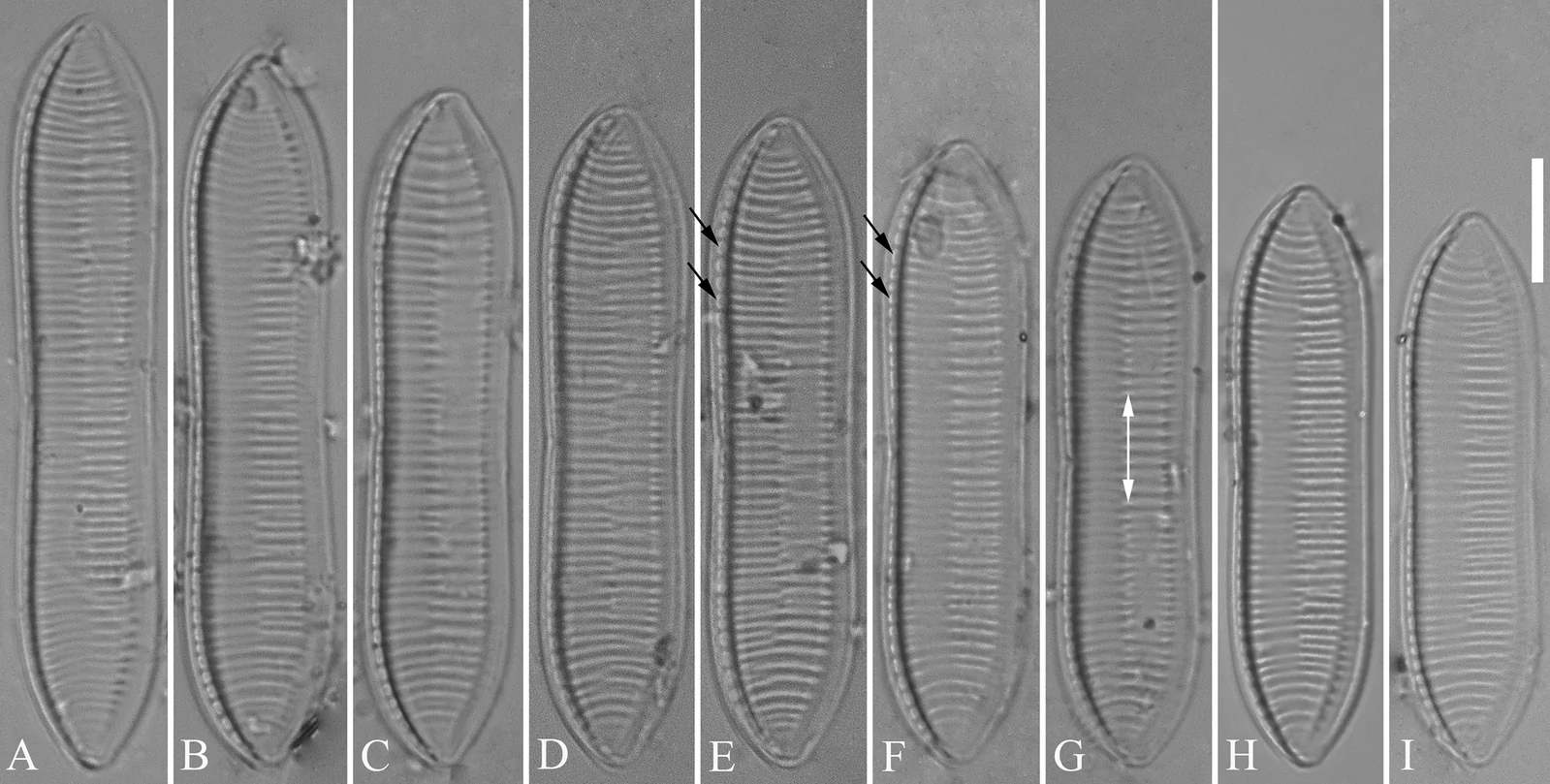
*Tryblionella dongtingensis* sp. nov., LM. **A–I**. Nine valves showing a size-diminution series; note the lack of an axial sternum, rectangular fibulae (e.g., **E**, **F**, arrows), and a longitudinal fold along the valve midline (e.g., **G**, double-headed arrow). **A**. Micrograph of the holotype specimen. Scale bar: 10 μm (**A–I**).

Stroganov (1927) made the valid transfer of *Nitzschia calida* Grunow (in Cleve and Grunow 1880) to *Tryblionella* before Mann (in Round et al. 1990). Therefore, the combination *Tryblionella calida* (Grunow) D.G. Mann should not be used.

### Tryblionella dongtingensis

Taxon classificationPlantaeBacillarialesBacillariaceae

Bing Liu & Rioual sp. nov.

F32B08FA-71E1-52C9-8764-F400FD061EF6

Figs 5, 6, 7

#### Holotype.

The specimen circled on the slide DIA2026002, illustrated here as Fig. 5A, deposited in the Herbarium of Jishou University (JIU), China. Registration: http://phycobank.org/106074.

**Figure 6.**
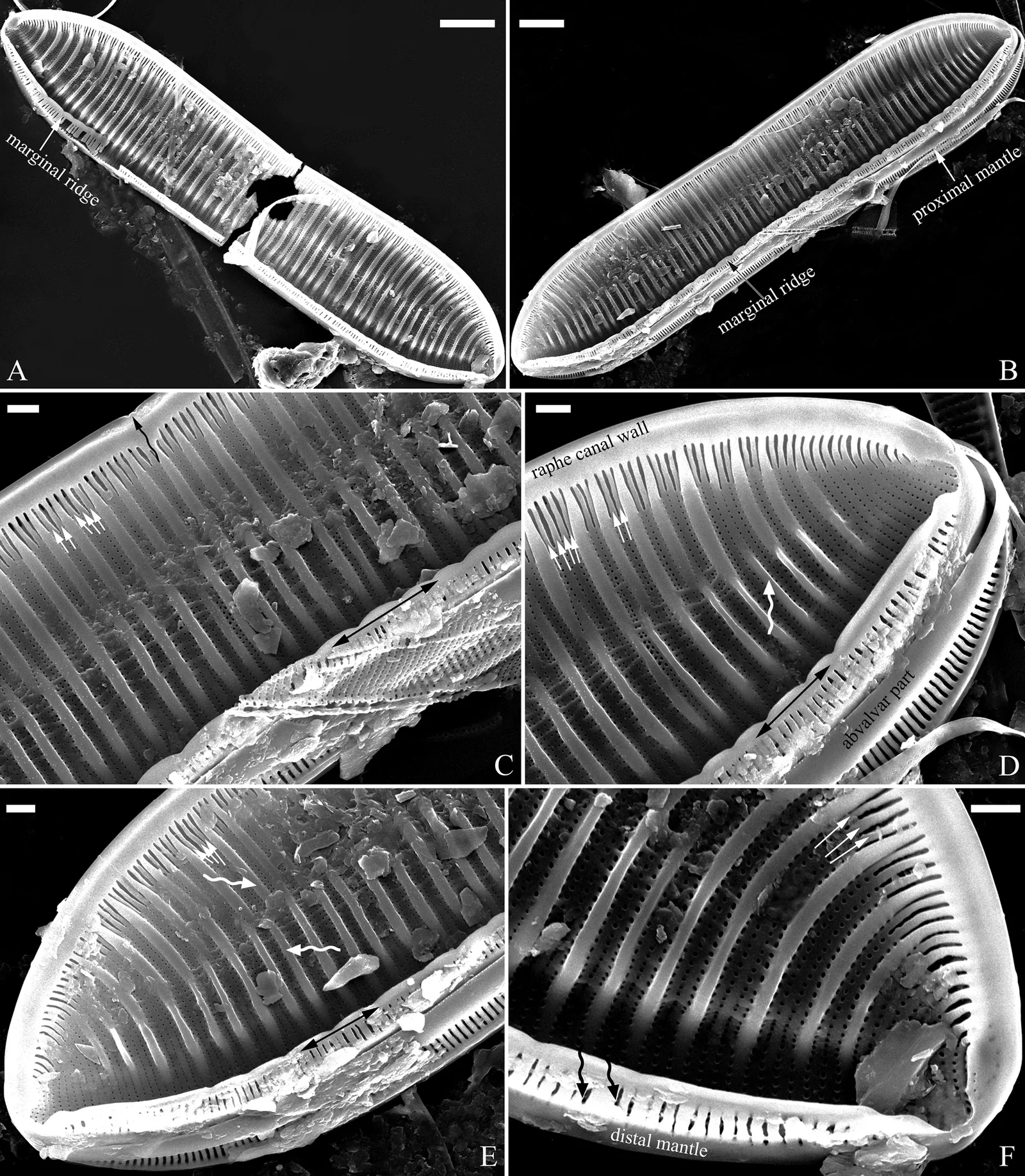
*Tryblionella dongtingensis* sp. nov., external view, SEM. **A**. A valve; note the high marginal ridge. **B**. A frustule; note the high marginal ridge and deep proximal mantle. **C–F**. Details from **B**; note that some transapical ribs are continuous across the valve face, whereas others terminate at the longitudinal fold (**D**, **E**, curved arrows), the uniseriate striae, the marginal ridge with very long slit-like fenestrae (**F**, two curved arrows) and an undulate top margin (**C–E**, black double-headed arrow), the bar-like ribs connecting the raphe canal wall (arrows), and the shallow distal mantle **F**. Scale bars: 5 μm (**A**, **B**); 1 μm (**C–F**).

**Figure 7.**
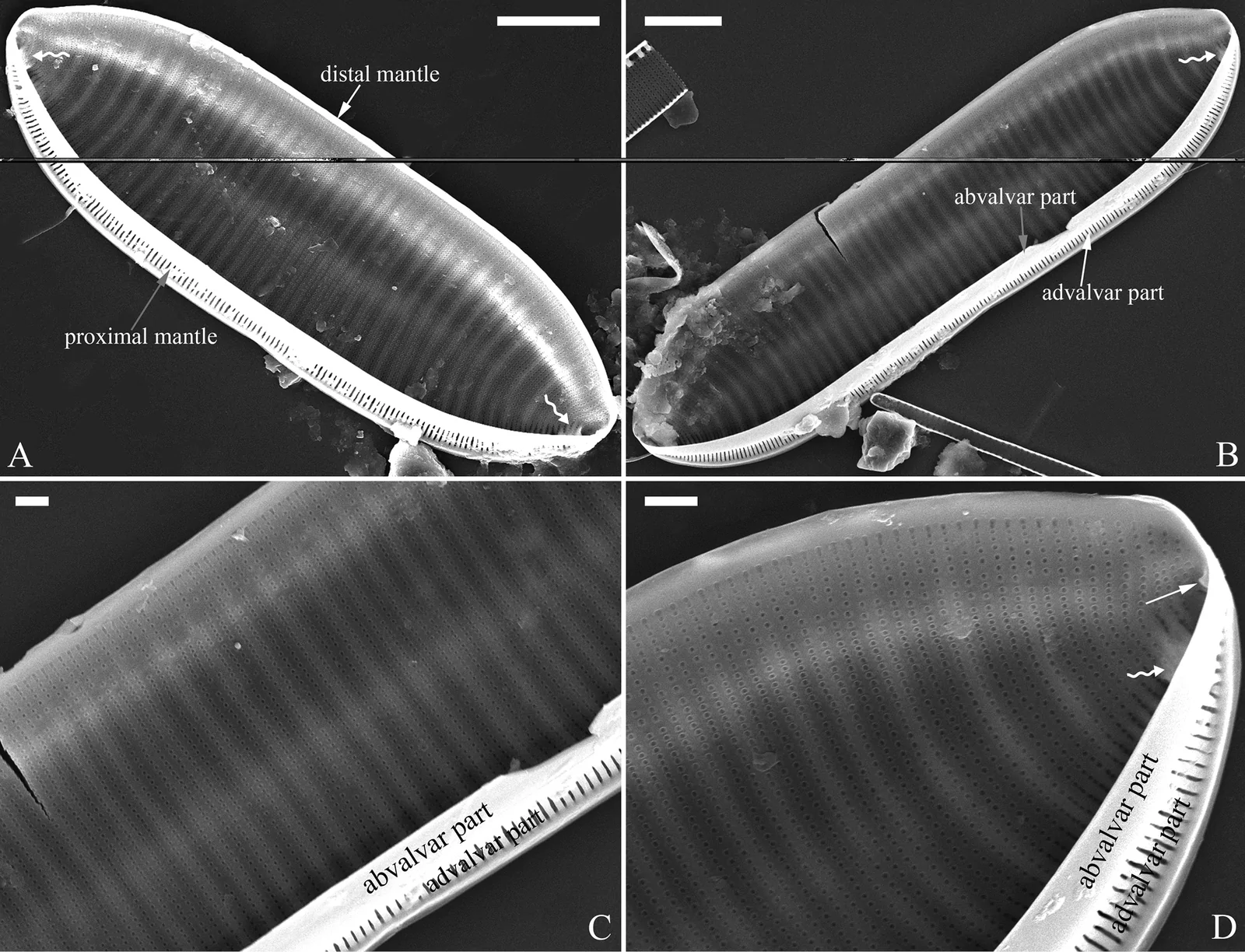
*Tryblionella dongtingensis* sp. nov., internal view, SEM. (**A**, **B**. Two complete valves; note that the markedly deep proximal mantle hides the fibulae and is composed of two different parts: an advalvar part and an abvalvar part. **C**, **D**. Details from **B**; note the rimmed, rounded inner openings of areolae, the advalvar part composed of bar-like ribs and narrow, rectangular openings, the abvalvar part lacking ornamentation, squat fibulae (**A**, **B**, **D**, curved arrow), and a small helictoglossa (**D**, arrow). Scale bars: 5 μm (**A**, **B**); 1 μm (**C**, **D**).

#### Type locality.

China • Hunan Province, East Dongting Lake. Sampling location (29°19'7"N, 112°47'32"E, 39 m a.s.l.) in the lake littoral zone, collected by Bing Liu, 5 March 2023.

#### Description.

***LM*** (Fig. 5). Valves linear-lanceolate, slightly constricted in the middle on both sides, with wedge-shaped apices. Valve dimensions (*n* = 30): length range 33–65 μm, width range 10.3–12.1 μm at constricted middle parts. Valve surface undulated from side to side. Axial sternum absent. A longitudinal fold is present along the valve midline (e.g., Fig. 5G, double-headed arrow), dividing the valve face into two non-coplanar halves. Transapical ribs more evident on one half of valve surface than on the other when observed at a given focal plane, 9–14 in 10 μm, parallel except approaching apices, where they become radiate. Raphe system eccentric, located along one side of valve only. Fibulae visible, short, rectangular (e.g., Fig. 5E, F, arrows), 6–9 in 10 μm.

***SEM*** (Figs 6, 7). Transapical ribs elevated, some run across the valve face continuously whereas others terminate at the middle longitudinal fold (Fig. 6D, E, curved arrows). Striae uniseriate, 38–40 in 10 μm (measured on SEM images, *n* = 3). A longitudinal row of bar-like ribs produced along the raphe canal wall, from pole to pole (Fig. 6C–F, arrows). Central nodule present (Fig. 6C, curved arrow). Distal raphe fissures curved (Fig. 6D). Marginal ridge is a high standing silica lamella, composed of a bottom part with long slit-like fenestrae (Fig. 6F, two curved arrows) and a top part with a serrate margin (Fig. 6C–E, double-headed arrows). Distal mantle very shallow, lacking ornamentations (Fig. 6F). Inner openings of areolae rounded, rimmed (Fig. 7D). Proximal mantle markedly deep so that it often hides the short fibulae (Fig. 7A). Proximal mantle composed of two parts: advalvar part (composed of bar-like ribs and narrow, rectangular openings), and abvalvar part (hyaline without ornamentations). Fibulae squat, often hidden by the deep proximal mantle (Fig. 7A, B, D, curved arrow), helictoglossa small (Fig. 7D, arrow).

#### Etymology.

Named after Dongting Lake, where the species was found.

#### Vernacular name.

The Chinese name for *Tryblionella dongtingensis* is “洞庭湖盘杆藻.”

#### Ecology and distribution.

*Tryblionella dongtingensis* sp. nov. was collected from the surface sediments in the littoral zone of East Dongting Lake. Associated species included *Craticula ambigua* D.G. Mann, *C. cuspidata* D.G. Mann, *Dorofeyukea kotschyi* Kulikovskiy, Kociolek, Tusset & T. Ludwig, *Gyrosigma dongtingense* Bing Liu & Rioual, *G. kuetzingii* (Grunow) Cleve, *Hantzschia amphioxys* Grunow, *Surirella angusta* Kützing, *S. suecica* Grunow, *Tryblionella calida*, *T. hungarica* (Grunow) Frenguelli, *Ulnaria rhombus* D.M. Williams, and several species of *Navicula* Bory, *Nitzschia* Hassall, and *Sellaphora* Mereschkowsky. The following environmental parameters were measured in the field: conductivity was 494 ± 0.9 μS cm^-1^, pH was 8.4 ± 0.06, and water temperature was 27.8 ± 0.3 °C. The environmental variables and the composition of the diatom assemblage suggest alkaline and mesotrophic to eutrophic conditions. So far, *T. dongtingensis* has been found only at the type locality.

#### Comments.

*Tryblionella dongtingensis* sp. nov. is characterized by its linear-lanceolate valve outline with wedge-shaped apices, undulate valve surface from side to side, elevated transapical ribs and short bar-like ribs connecting the raphe canal wall, uniseriate striae, short fibulae, and a high marginal ridge composed of a bottom part with elongate slit-like fenestrae and a solid top part with an undulate margin. A review of the literature revealed few species similar to *T. dongtingensis* sp. nov. The most similar species to *T. dongtingensis* is *T. calida*, and they can coexist in the same habitat. The two species, however, differ in their morphometry. *Tryblionella calida* has narrower valves (8.6–10.6 μm vs. 10.3–12.1 μm), a higher density of transapical ribs (15–17 in 10 μm vs. 9–14 in 10 μm), and a higher density of fibulae (9–10 in 10 μm vs. 6–9 in 10 μm). In addition, *T. calida* has almost flat valve surfaces (Fig. 1), whereas *T. dongtingensis* has an undulate valve surface (Fig. 5). Furthermore, the marginal ridge in *T. calida* has a plain top margin (see Fig. 2), whereas in *T. dongtingensis* it has an undulate top margin (see Fig. 6).

### Tryblionella hunanensis

Taxon classificationPlantaeBacillarialesBacillariaceae

Bing Liu & Rioual sp. nov.

B874CEDB-D961-506B-A878-9FBFC975CA9E

**Figure 8.**
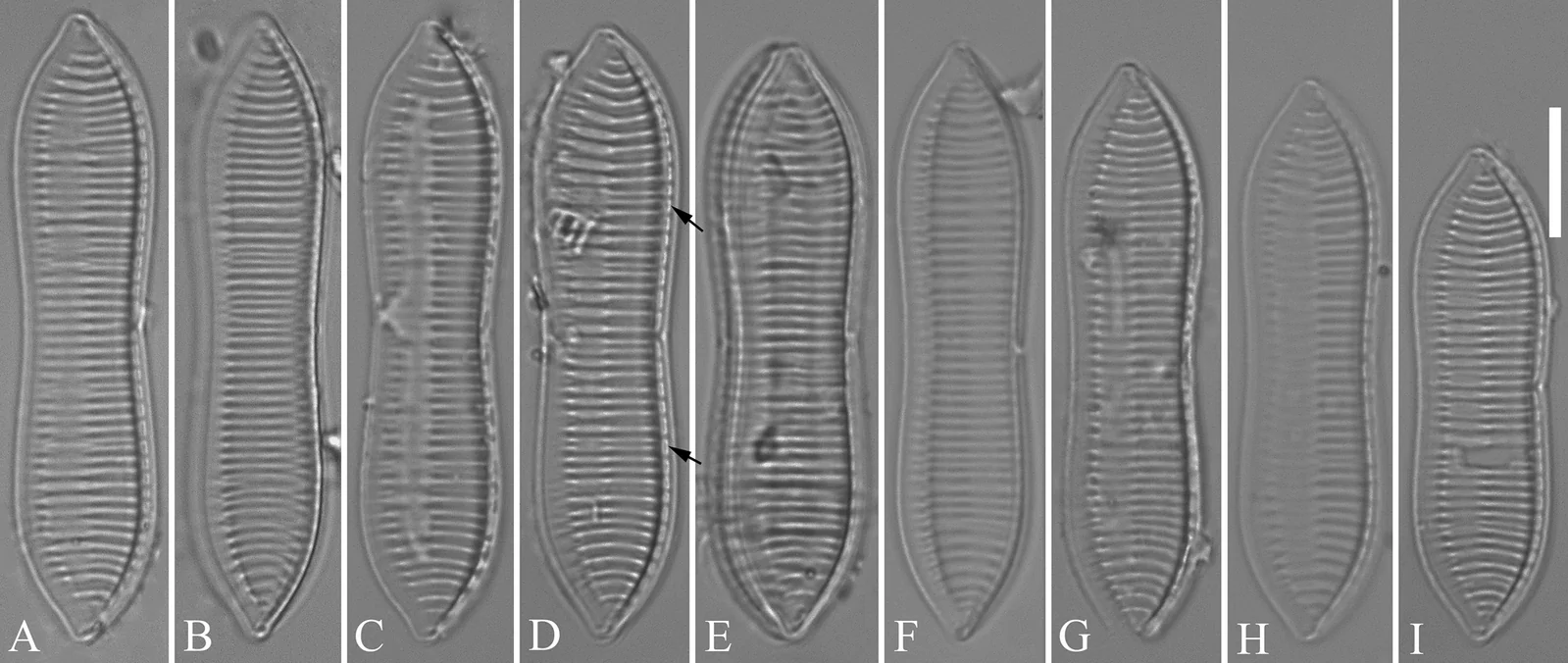
*Tryblionella hunanensis* sp. nov., LM. **A–I**. Nine valves showing a size-diminution series; note the absence of an axial sternum and rectangular fibulae (e.g., **D**, two arrows). **A**. Micrograph of the holotype specimen. Scale bar: 10 μm (**A–I**).

**Figure 9.**
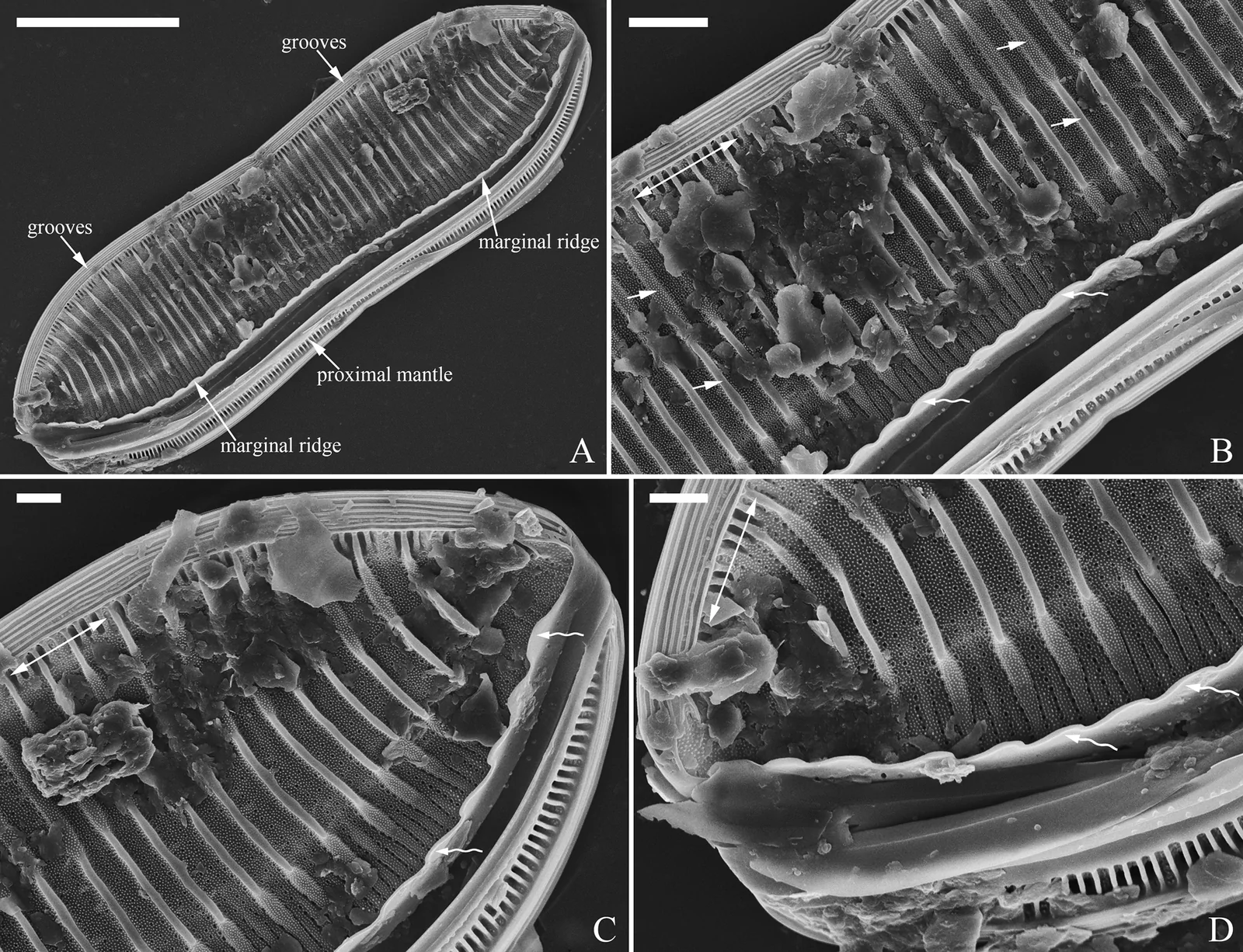
*Tryblionella hunanensis* sp. nov., SEM. **A**. A disarticulated frustule; note the high marginal ridge, deep proximal mantle, and longitudinal grooves in the raphe canal wall on the side of the valve surface. **B–D**. Details from **A**; note the dense silica granules produced on the valve surface and on parts of the transapical ribs on the raphe canal side, whereas the parts of the transapical ribs on the side of the marginal ridge are not covered with these silica granules (**B**, arrows), a longitudinal row of bar-like ribs connecting the raphe canal wall (double-headed arrows), and the distinctly lobed top margin of the marginal ridge (curved arrows). Scale bars: 10 μm (**A**); 2 μm (**B**); 1 μm (**C**, **D**).

**Figure 10.**
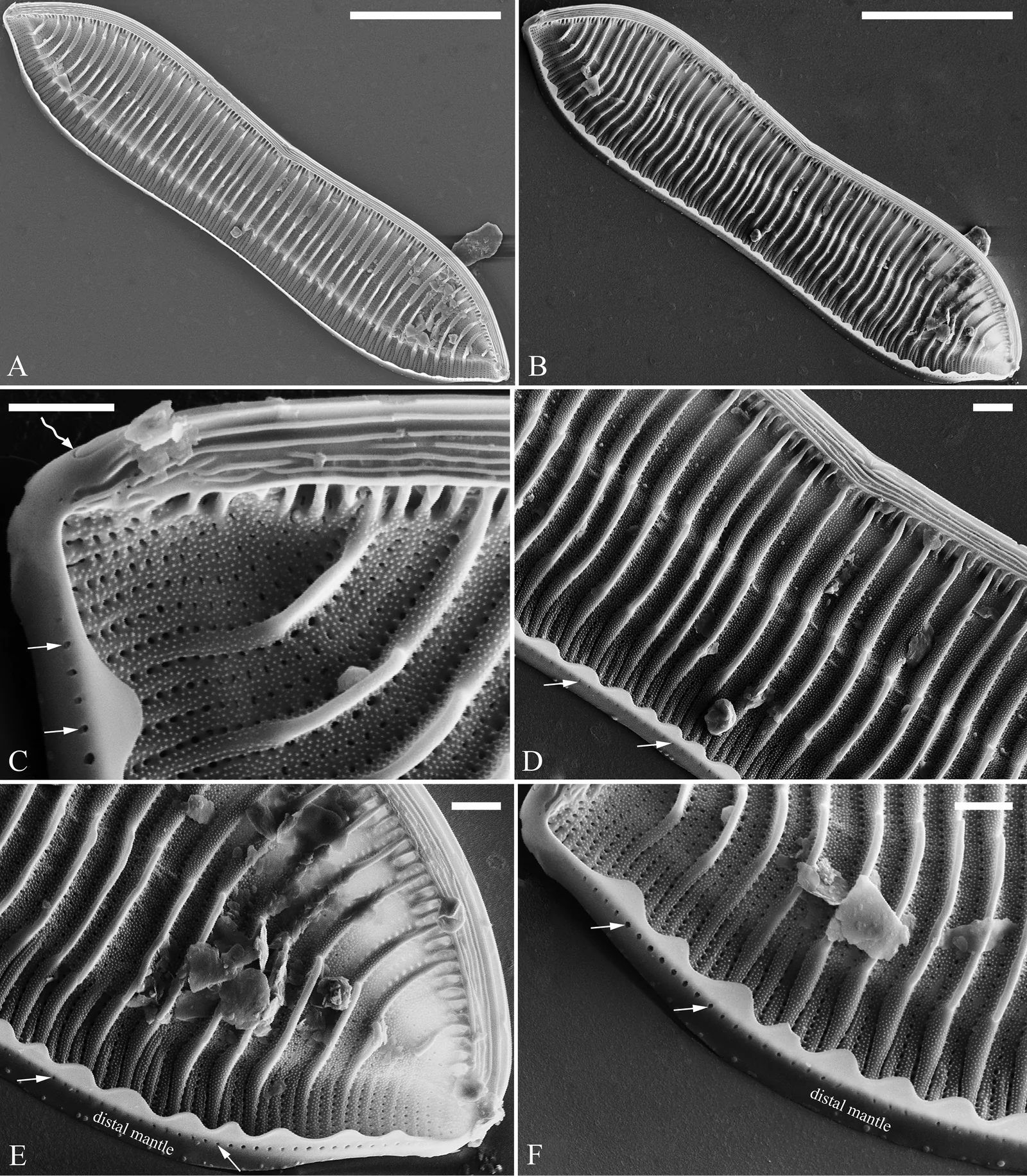
*Tryblionella hunanensis* sp. nov., external view, SEM. **A**, **B**. Two valves; note the distinct transapical ribs, high marginal ridge, and grooves in the raphe canal wall. **C–F**. Details from **B**; note the lobed top margin of the marginal ridge, a row of pores produced along the junction between the distal mantle and the base of the marginal ridge (arrows), and a curved distal raphe fissure (**C**, curved arrow). Scale bars: 10 μm (**A**, **B**); 1 μm (**C–F**).

**Figure 11.**
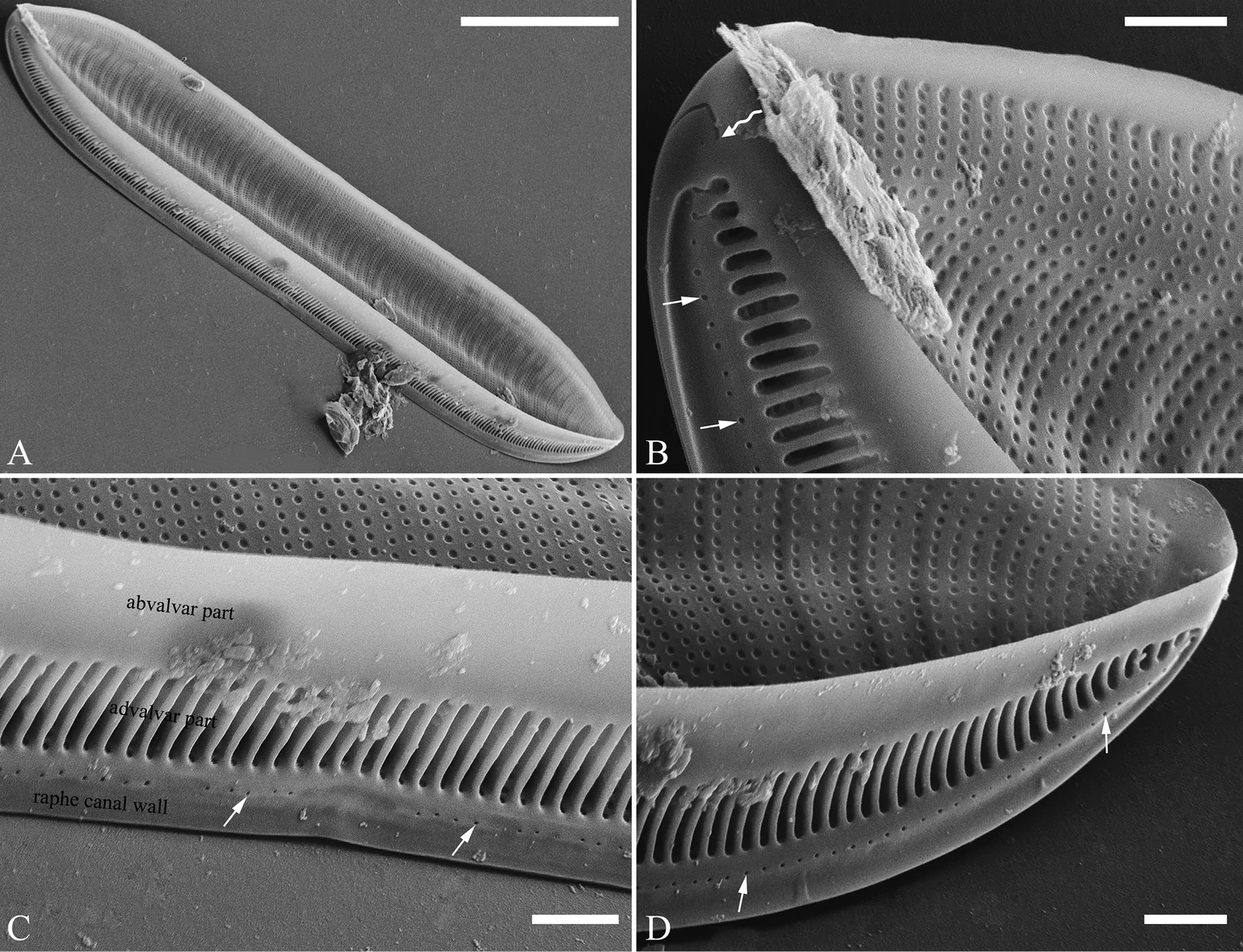
*Tryblionella hunanensis* sp. nov., proximal mantle aspect, SEM. **A**. A valve; note the very deep proximal mantle. **B–D**. Details from **A**; note that the proximal mantle is composed of two parts: an advalvar part composed of bar-like ribs and a hyaline abvalvar part, and a row of poroids is produced in the raphe canal wall on the side of the proximal mantle (arrows). Scale bars: 10 μm (**A**); 1 μm (**B–D**).

**Figure 12.**
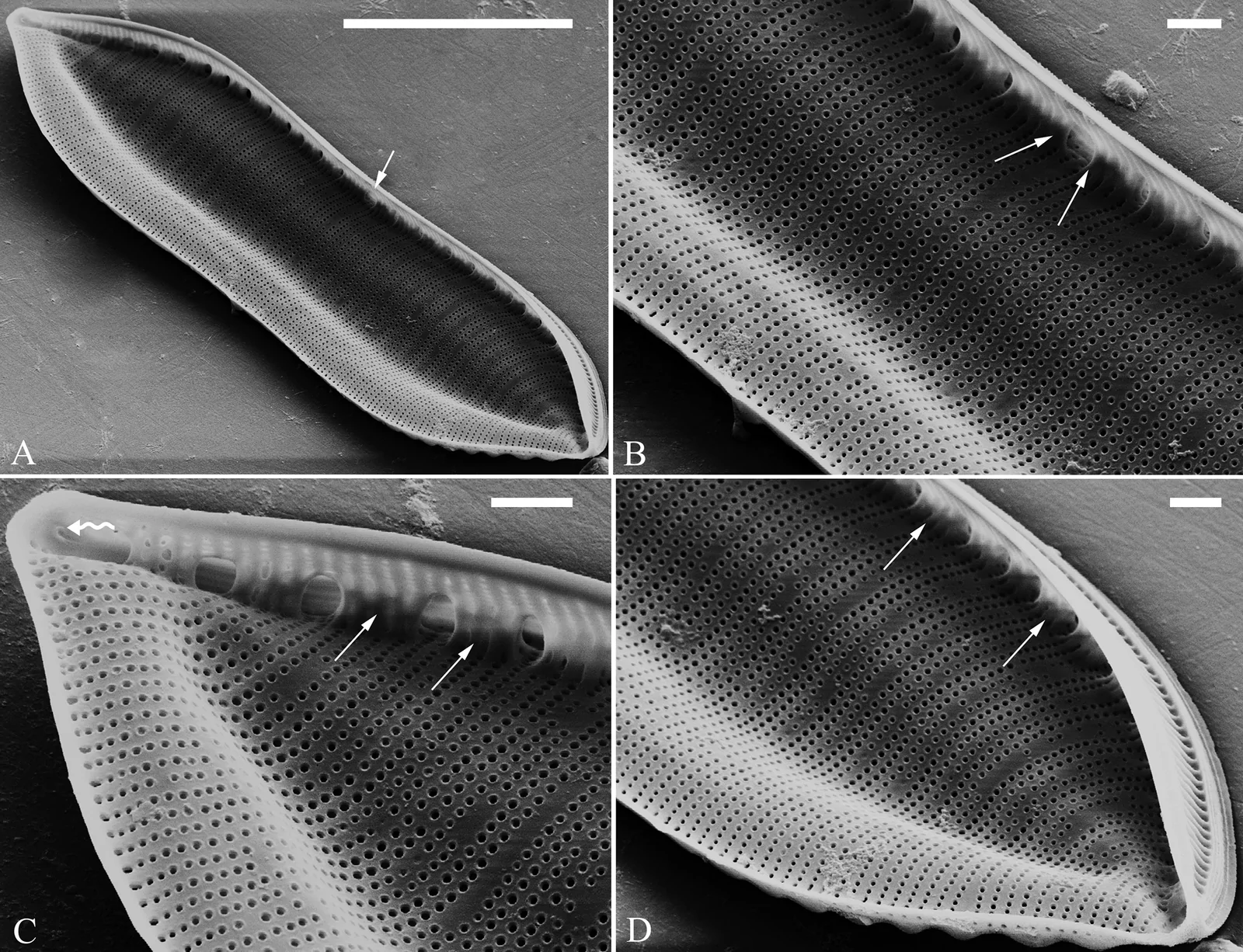
*Tryblionella hunanensis* sp. nov., internal view, SEM. (**A**). A complete valve; note that the two median fibulae are farther apart (arrow). **B–D**. Details from **A**; note the rimmed, rounded inner openings of areolae, the two median fibulae farther apart (**B**, two arrows), squat, mostly apically elongated fibulae (**C**, **D**, arrows), and a small helictoglossa (**C**, curved arrow). Scale bars = 10 μm (**A**), 1 μm (**B–D**).

Figs 8, 9, 10, 11, 12

#### Holotype.

The specimen circled on the slide DIA2026003, illustrated here as Fig. 8A, deposited in the Herbarium of Jishou University (JIU), China. Registration: http://phycobank.org/106075.

#### Type locality.

China • Hunan Province, Yongle River (a tributary of the largest river, Xiang River, in Hunan Province, China). Sampling location (26°55'08"N, 113°35'55"E, 119 m a.s.l.) in a riffle, collected by Bing Liu, 24 March 2024.

#### Description.

***LM*** (Fig. 8). Valves panduriform with cuneate to acute apices. Valve dimensions (*n* = 31): length range 37–53 μm, width range 8.9–13.2 μm at constricted middle parts. Valve surfaces slightly undulated from side to side. Axial sternum absent. Transapical ribs parallel, across valve face continuously, 9–12 in 10 μm. Raphe system eccentric, located only along one side of valve. Fibulae visible, rectangular to square (e.g., Fig. 8D, two arrows), 7–10 in 10 μm.

***SEM*** (Figs 9, 10, 11, 12). Valves panduriform with elevated transapical ribs continuously across valve surface (Figs 9, 10). Dense silica granules present on the valve surface, striae uniseriate, 34–36 in 10 μm (measured on SEM images, *n* = 3). A longitudinal row of bar-like ribs produced along the raphe canal wall, from pole to pole (Fig. 9B–D, double-headed arrows). Marginal ridge is a standing silica lamella, composed of a bottom part with a row of pores produced along the junction between the distal mantle and the base of the marginal ridge, and a solid top part with a distinctly lobed margin (Figs 9, 10). Four to seven longitudinal grooves are produced in the raphe canal wall on the side of valve (Figs 9A, 10A, 10B). Distal mantle shallow, lacking ornamentations (Fig. 10C–F). Distal raphe fissures curved (Figs 10C, 11B, curved arrows). Proximal mantle markedly deep, composed of two parts: advalvar part (composed of bar-like ribs and narrow, rectangular openings), and abvalvar part (hyaline part without ornamentations) (Fig. 11). A row of poroids produced in the raphe canal wall on the side of the proximal mantle (Fig. 11B–D, arrows). Inner openings of areolae rounded, rimmed. Fibulae squat, mostly apically elongated (Fig. 12), two median fibulae farther apart (Fig. 12A, B, arrows). Helictoglossae small (Fig. 12C, D, curved arrows).

#### Etymology.

Named after Hunan Province, where the species was found (specific locality is Yongle River).

#### Vernacular name.

The Chinese name for *Tryblionella hunanensis* is “湖南盘杆藻.”

#### Ecology and distribution.

*Tryblionella hunanensis* sp. nov. was collected from the mud sediments on the stone surfaces in the Yongle River. There are numerous associated species, with the most abundant being *Ctenophora hunanensis* Bing Liu & Rioual, *Cymbella turgidula* Grunow, *Grunowia tabellaria* (Grunow) Rabenhorst, *Gyrosigma kuetzingii* (Grunow) Cleve, *Hippodonta capitata* (Ehrenberg) Lange-Bertalot, Metzeltin & Witkowski, *Nitzschia palea* (Kützing) W. Smith, *Tryblionella calida*, *Ulnaria fanjingensis* Bing Liu, and several species of *Cocconeis* Ehrenberg, *Fragilaria* Lyngbye, *Gomphonella* Rabenhorst, *Luticola* D.G. Mann, *Navicula* Bory, *Planothidium* Round & Bukhtiyarova, *Sellaphora* Mereschowsky, and *Surirella* Turpin. The following environmental parameters were measured in the field: conductivity was 111.7 ± 0.8 μS cm^–1^, pH was 7.6 ± 0.04, and water temperature was 22.3 ± 0.3 °C. The environmental variables and the composition of the diatom assemblage suggest alkaline and mesotrophic conditions. So far, *T. hunanensis* has been found at the type locality and in East Dongting Lake.

#### Comments.

**Figure 13.**
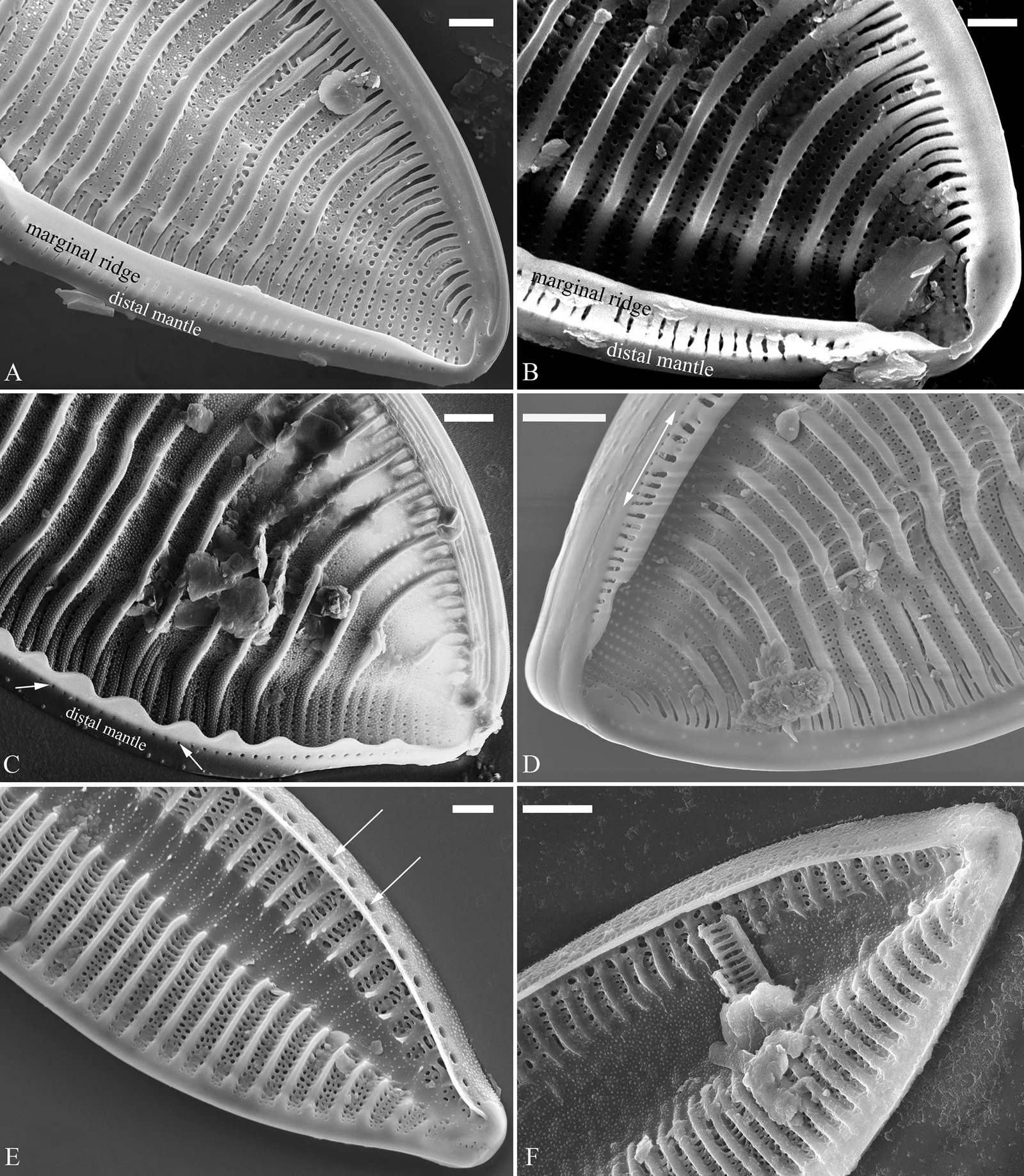
Marginal ridges in six species of *Tryblionella*, external view, SEM. **A**. *T. calida*. **B**. *T. dongtingensis* sp. nov. **C**. *T. hunanensis* sp. nov. **D**. *T. levidensis*. **E**. *T. apiculata*. **F**. *T. qinghainensis*. Scale bars: 1 μm (**A–C**, **E**); 2 μm (**D**).

*Tryblionella hunanensis* sp. nov. is characterized by its panduriform valve outline with cuneate to acute apices, slightly undulate valve surface, elevated transapical ribs continuous across the valve face and short bar-like ribs connecting the raphe canal wall, uniseriate striae, squat, mostly apically elongated fibulae, and a marginal ridge composed of a bottom part with a row of pores produced along the junction between the distal mantle and the base of the marginal ridge and a solid top part with a distinctly lobed margin. A review of the literature revealed few species similar to *T. hunanensis* sp. nov. The species most similar to *T. hunanensis* are *T. calida* and *T. dongtingensis*. All three species have similar valve outlines and overlapping morphometric ranges, and they can coexist in the same habitat in East Dongting Lake. However, *T. calida* has almost flat valve surfaces (Fig. 1), whereas *T. hunanensis* has a more undulate valve surface (Fig. 13). Furthermore, the solid top part of the marginal ridge in *T. calida* is straight (see Fig. 2), whereas in *T. hunanensis* it is distinctly lobed (see Fig. 14). *Tryblionella hunanensis* also differs from *T. dongtingensis* in the structure of the raphe canal wall on the valve side (in the former, it is grooved, whereas in the latter, it is smooth) and the marginal ridge (in the former, its bottom part has no slit-like fenestrae, whereas slit-like fenestrae are present in the latter).

## Discussion

### Marginal ridge in *Tryblionella*

When Round et al. (1990) resurrected the genus *Tryblionella*, they pointed out that its valve face often bears a marginal ridge at its junction with the extremely shallow distal mantle. These authors also mentioned that the marginal ridge can be high or low and perforated or solid. However, as reviewed and summarized by Ma et al. (2025), the marginal ridge has often been neglected in the published literature on *Tryblionella* since 1990 and has rarely been mentioned or illustrated, let alone described. Ma et al. (2025) described the marginal ridges of three *Tryblionella* species found in the brackish-water habitat of Lake Qinghai and observed that the structures of the marginal ridges and their junctions with the distal mantles are highly variable and species-specific. These findings suggested that the marginal ridge can be an important character for delimiting different species of *Tryblionella*.

Interestingly, the three species of *Tryblionella* described in this paper all bear a marginal ridge. Furthermore, the marginal ridges of *T. calida* and *T. dongtingensis* sp. nov. possess similar structures: a high-standing silica lamella composed of a bottom part with long slit-like fenestrae and a solid top part with a plain margin (Fig. 13A, B), whereas the marginal ridge of *T. hunanensis* sp. nov. has a different structure: a standing silica lamella composed of a bottom part with a row of pores produced along the junction between the distal mantle and the base of the marginal ridge and a solid top part with a distinctly lobed margin (Fig. 13C). The marginal ridge of *T. levidensis* is like that of *T. calida*, except for an additional longitudinal row of poroids produced along the junction between the marginal ridge and distal mantle (Fig. 13D, double-headed arrow). The marginal ridge of *T. apiculata* is a standing, solid silica lamella with a plain top margin and a row of large pores produced along the junction between the marginal ridge and the distal mantle (Fig. 13E, two arrows). The marginal ridge of *T. qinghainensis* is a standing, solid silica lamella (Fig. 13F).

Olszyński et al. (2025) also reviewed the marginal ridge in *Tryblionella* and proposed four types of marginal ridges: marginal ridge absent or indistinct, non-continuous ridge, piping ridge, and sail-like ridge. Based on the above analysis of marginal ridges, the structural diversity of the marginal ridge is evident in the following aspects: 1) the marginal ridge is solid or perforated; if perforated, the shape and position of the perforations can be variable; 2) the top margin is continuous or interrupted and can be smooth or not (e.g., undulate, serrated, or lobed); and 3) a row of pores or poroids is present or absent along the junction between the base of the marginal ridge and distal mantle.

### Group around *Tryblionella calida*

The three species of *Tryblionella* described in this paper all occur in freshwater habitats. In addition, they have a unique combination of characters: 1) a more or less undulate valve face from side to side; 2) transapical ribs; 3) uniseriate striae with rounded, rimmed inner openings of areolae; 4) a longitudinal row of bar-like ribs connecting the raphe canal wall on both the valve side and the distal mantle side; 5) a marginal ridge; and 6) squat, sometimes apically elongated fibulae. The group around *T. apiculata* has an axial sternum and striae and areolae that differ from those of the group of *T. calida*, *T. dongtingensis* sp. nov., and *T. hunanensis* sp. nov. Thus, morphologically, *T. calida*, *T. dongtingensis* sp. nov., and *T. hunanensis* sp. nov. can be regarded as a distinct group within *Tryblionellasensu lato*.

Olszyński et al. (2025) established a majority-rule consensus tree (based only on selected morphological characters and character states) in which there is a clade—Clade IB [including six species, *T. calida*, *T. circumsuta* (Bailey) Ralfs, *T. ornata* Bertolli, Talgatti & Torgan, *T. debilis* Arnott ex O’Meara, *T. confusa* Bertolli & Torgan, and *T. victoriae* Grunow]—and proposed a possible synapomorphy for the taxa within Clade IB: the presence of perforated fibulae. However, Olszyński et al. (2025) also noted that the nature of the fibulae in *T. confusa* and *T. calida* had not yet been confirmed. In this paper, it is shown for the first time that the fibulae of *T. calida* are non-perforated (Fig. 4). Thus, whether the group around *T. calida* is monophyletic and possesses a synapomorphy cannot currently be resolved, and further investigations are needed.

## Supplementary Material

XML Treatment for Tryblionella calida

XML Treatment for Tryblionella dongtingensis

XML Treatment for Tryblionella hunanensis

## Additional information

### Artificial Intelligence (AI) use

The authors accept full responsibility for the content of the manuscript, including the disclosure of any use of AI.

Regarding the use of AI in the preparation of this manuscript, the authors declare the following: GPT

Used for: Language, style and writing

We have used GPT to modify the English language of this submitted manuscript.

### Author ORCIDs

Y.-L. Ma https://orcid.org/0009-0003-7838-3494

B. Liu https://orcid.org/0000-0002-8516-325X

P. Rioual https://orcid.org/0000-0001-9491-9197

X.-Y. Jiang https://orcid.org/0009-0004-6760-9555

### Data availability

All of the data that support the findings of this study are available in the main text.
